# Optimal conditions of algal breeding using neutral beam and applying it to breed *Euglena gracilis* strains with improved lipid accumulation

**DOI:** 10.1038/s41598-024-65175-1

**Published:** 2024-07-03

**Authors:** Sousuke Imamura, Koji Yamada, Hiroaki Takebe, Ryu Kiuchi, Hidenori Iwashita, Chihana Toyokawa, Kengo Suzuki, Atsushi Sakurai, Kazuhiro Takaya

**Affiliations:** 1 Space Environment and Energy Laboratories, Nippon Telegraph and Telephone Corporation, Musashino-shi, 180-8585 Japan; 2 Advanced Science Research Institute, Euglena Co., Ltd., Yokohama-shi, 230-0045 Japan

**Keywords:** Algal oil, Neutron beam, Microalga, Mutagenesis, Strain development, Plant breeding, Biological techniques, Genetic techniques

## Abstract

Microalgae are considered to be more useful and effective to use in biomass production than other photosynthesis organisms. However, microalgae need to be altered to acquire more desirable traits for the relevant purpose. Although neutron radiation is known to induce DNA mutations, there have been few studies on its application to microalgae, and the optimal relationship between irradiation intensity and mutation occurrence has not been established. In this study, using the unicellular red alga *Cyanidioschyzon merolae* as a model, we analyzed the relationship between the absorbed dose of two types of neutrons, high-energy (above 1 MeV) and thermal (around 25 meV) neutrons, and mutation occurrence while monitoring mutations in *URA5.3* gene encoding UMP synthase. As a result, the highest mutational occurrence was observed when the cells were irradiated with 20 Gy of high-energy neutrons and 13 Gy of thermal neutrons. Using these optimal neutron irradiation conditions, we next attempted to improve the lipid accumulation of *Euglena gracilis*, which is a candidate strain for biofuel feedstock production. As a result, we obtained several strains with a maximum 1.3-fold increase in lipid accumulation compared with the wild-type. These results indicate that microalgae breeding by neutron irradiation is effective.

## Introduction

Microalgae produce biomass through photosynthesis using sunlight, water, and carbon dioxide. Many microalgae generally produce biomass at higher rates than terrestrial plants and do not require arable land^1,2^. By cultivating Chlorella, Spirulina, and other microalgae on a large scale in low latitude regions, algal biomass has been commercially produced for food products^3^. The recent advances in cultivation technologies are lowering the production cost of algal biomass, leading to the attempts to expand the use of microalgae to bioactive substances, polysaccharides, and lipids as a biofuel ingredient^4^. However, to commercialize the new applications, productivity needs to be improved by modifying the inherent properties of microalgae.

One method for modifying the properties of microalgae is to create mutants using genetic recombination and genome editing technologies^5–7^. In this case, genome sequence information of the target microalgae is required, and the gene directly linked to the desired trait change must be identified in advance. In addition, to modify or edit the gene, molecules such as nucleic acids or proteins that modify the target gene from outside the cell need to be introduced. These have the limitation that they are not immediately applicable to all microalgae. A technique not subject to those limitations has been to randomly introduce mutations into genomic DNA and isolate strains that exhibit the desired trait from among them^8,9^. Mutations to DNA can be introduced by several methods including exposure to chemical mutagens such as ethane methyl sulfonate and *N*-methyl-*N*′-nitro-*N*-nitrosoguanidine and irradiation to light energy including ultraviolet radiation, low linear energy transfer (LET) rays such as gamma or X-rays, high-LET rays such as proton beams, heavy particle, and neutron beams^8,9^.

Neutron beams possess a high permeability property since they have no electric charge and are considered more suitable than the other methods for breeding via genomic DNA mutation to microalgae growing in liquid culture media. However, to assess the quality of mutagenesis, key parameters such as the mutation rate need to be monitored, but their general standardization has not yet been established and depends on the experience of the scientists and the laboratory infrastructure^9^. It is reported that lipid productivity is enhanced in green microalgae *Chlorella* sp. after exposure to neutron irradiation^10^. However, there have been no reports on the isolation of microalgal strains that show the desired property among the neutron-irradiated algal culture. Concerning neutrons themselves, high-energy neutrons (above 1 MeV) are the main component when neutrons are produced^11^, so high-energy neutrons can be moderated to thermal neutrons (around 25 meV) by using a hydrogen-containing material as a moderator^12^. However, the effects of these two different neutron beams on the introduction of mutations into microalgae DNA are not clear.

In this study, we clarified the relationship between absorbed intensities of high-energy/thermal neutrons and mutation occurrence in the gene encoding uracil synthase^13^ in the unicellular red alga *Cyanidioschyzon merolae*, one of the model algae^14^. Furthermore, we also clarified how the two types of neutron irradiation affect the physiological process by monitoring transcript levels. To demonstrate the general effect of various algal species, we also evaluated the effect of neutron irradiation on *Euglena gracilis*, which is a candidate strain for biofuel feedstock production^15,16^. *E. gracilis*, which belongs to Excavata, is highly divergent from other eukaryotic algae in Plantae, shows resistance to mutagens, and has been successfully bred only by using Fe-ion beam as a mutagen^17–19^. By utilizing the underlying information of neutron irradiation conditions against *C. merolae* culture, we succeeded in isolating four mutant strains of *E. gracilis*, with a maximum 1.3-fold increase in lipid accumulation. These results clearly indicate that algal breeding by neutron irradiation is effective.

## Results

### Optimal neutron absorption dose for mutation induction in algae

We evaluated the relationship between neutron irradiation intensity and mutagenesis in algal genomic DNA to determine the optimal absorbed dose for mutagenesis in genomic DNA by neutron irradiation. In the evaluation, the introduction of mutations in the algal population irradiated with neutrons needs to be quantitatively evaluated. For this purpose, a method is needed to select strains in which mutations have been introduced. In addition, haploid algae are ideal for easily identifying mutation sites and characterizing their style, using sequence analysis, as a first step. In this study, we chose the unicellular red alga *C. merolae* as the target of our analysis. *C. merolae* possesses haploid genomic DNA^14^, and an experimental method has been reported to isolate strains of mutations introduced into the uracil synthesis gene by using the uracil requirement and 5-fluoroorotic acid (5-FOA) resistance as indicators^13,20^.

**Figure 1:**
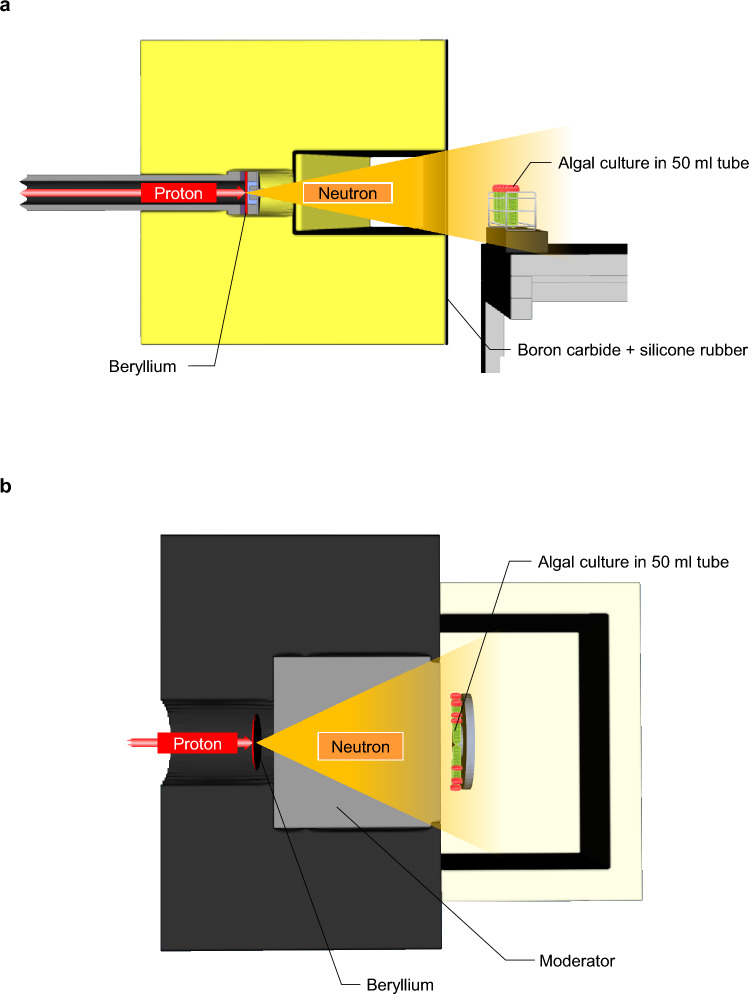
Neutron irradiation facilities. Panel (**a**) schematic representation of facility for high-energy neutron beams. Boron carbide + silicone rubber was placed to reduce thermal neutrons. 50 mL tubes containing algal culture were located at an indicated distance from the neutron production target. Panel (**b**) schematic representation of facility for thermal neutron beams. Moderator was installed to slow down the passing high-energy neutrons and convert them to thermal neutrons. 50 mL tubes containing algal culture were attached to the jig, which rotated at 2 rpm during the irradiation.

Since neutron beams can be divided into high-energy neutrons and thermal neutrons, the effects of the two types of neutron beams on algal mutagenesis and the optimal irradiation conditions for each were individually investigated. Figure 1 shows a schematic diagram of the two types of neutron beam irradiation toward algal culture: panel a shows high-energy neutrons, and panel b shows thermal neutrons.

**Figure 2:**
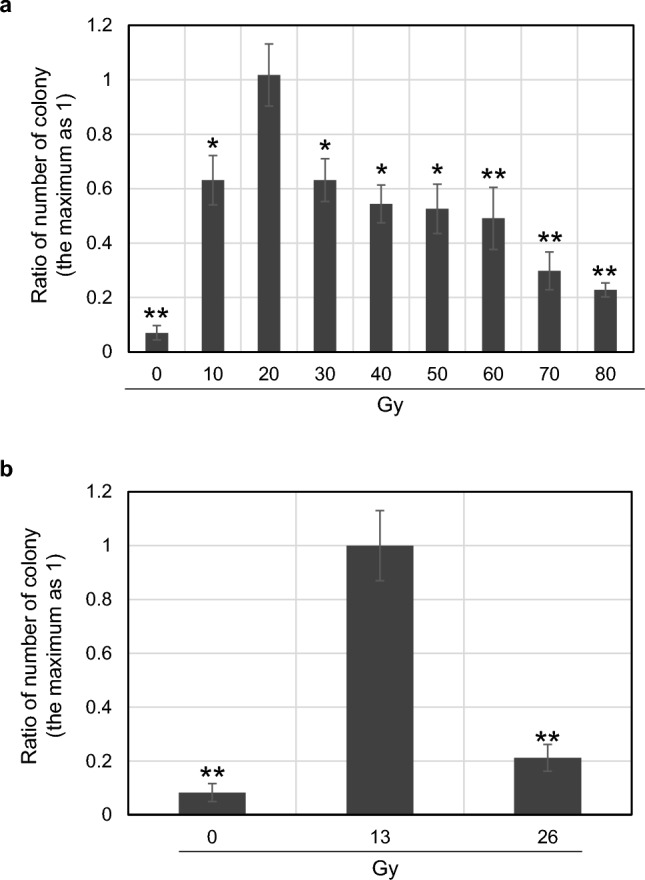
Relationship between neutron intensity and the appearance of 5-FOA resistance strain. Panel (**a**) relationship between high-energy neutrons and the appearance of 5-FOA resistance strain. Algal cultures were irradiated with high-energy neutron beams at indicated absorbed doses using the facility shown in Fig. 1a. The cells were then plated to the medium containing 5-FOA, and the number of resistant strain colonies was counted after a few weeks of incubation. Relative values are shown when the number with the highest appearance number is set to 1.0. n = 3, t-tests comparing the highest value (20 Gy) and the other ones; **p < 0.01; *p < 0.05. Panel (**b**) relationship between thermal neutrons and the appearance of 5-FOA resistant strains. The others are the same as panel (**a**), but algal cultures were irradiated with thermal neutrons by the facility shown in Fig. 1b.

The neutron-irradiated *C. merolae* cells were plated on a solid medium containing uracil and 5-FOA, and colony formation was observed. 5-FOA is converted to fluorodeoxyuridine, which is toxic to cells, by URA3 (URA5.3 in the case of *C. merolae*)^13^ encoding UMP synthase, which is essential for uracil synthesis for algal strains. Thus, strains harboring functional URA5.3 cannot survive in the medium containing 5-FOA. On the other hand, if a mutation(s) occurs in the *URA5.3* gene and causes loss or change of its protein function, fluorodeoxyuridine is not synthesized and the strain can survive on the plate containing 5-FOA and uracil. By using this mechanism, 5-FOA-resistant strains can be obtained to monitor the introduction of mutations into the *URA5.3* gene in the medium containing 5-FOA and uracil. A representative example of the results of 5-FOA-resistant colony selection, growing on a 5-FOA-containing solid medium, is shown in Fig. S1. The efficiency of mutation into the *URA5.3* gene was evaluated from the information on the number of 5-FOA-resistant colonies. Figure 2a shows the results of irradiation with high-energy neutron beams to an absorbed dose of 0–80 Gy, in 10 Gy increments. As the results show, in the case of high-energy neutron beams, most colonies appeared at 20 Gy, at which about 24 colonies per 1 × 10^8^ cells were observed. On the other hand, in the case of thermal neutrons, the number of colonies was higher at 13 Gy, at which about 28 colonies were observed, than at 0 and 26 Gy (Fig. 2b). The growth of irradiated *C. merolae* cells, which were transferred to a new medium, was not significantly different (Fig. S1), indicating that the difference in 5-FOA resistance was not due to the difference in cell viability after irradiation and its intensity. These results indicate that 20 Gy for high-energy neutrons and 13 Gy for thermal neutrons are suitable for introducing mutations into the *URA5.3* synthesis gene in *C. merolae*.

### Type of mutations introduced in the *URA5.3* gene

**Figure 3:**
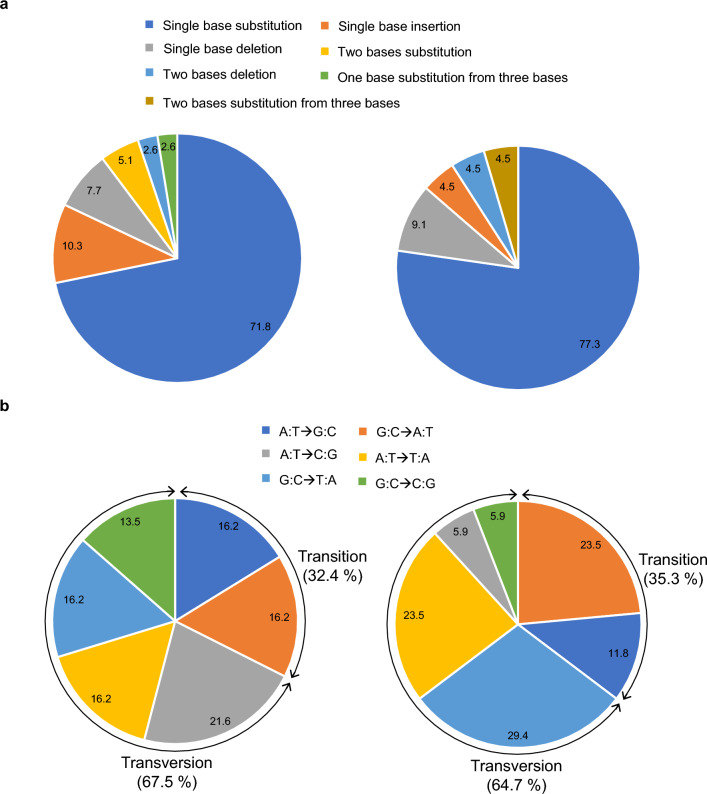
Mutation patterns detected in the *URA5.3* gene in 5-FOA-resistant strains. Panel (**a**) mutation patterns in 24 5-FOA-resistant strains obtained by high-energy neutron (left) and in 20 5-FOA-resistant strains obtained by thermal neutron (right) irradiation. The indicated mutation patterns are shown in %. Panel (**b**) characterization of single-base substitution. The single-base substitutions shown in panel (**a**) (28 and 17 sites in 5-FOA-resistant strains by high-energy and thermal neutron irradiation, respectively) are classified into transition and transversion, and their contents are indicated (left, high-energy neutron; right, thermal-energy neutron). The indicated number denotes % of each category.

We analyzed the type of mutations introduced to the *URA5.3* gene in 5-FOA-resistant strains. To this end, the region containing the *URA5.3* gene of strains isolated under the optimal neutron irradiation conditions, specifically 20 and 13 Gy of high-energy and thermal neutrons, respectively, was amplified by polymerase chain reaction (PCR), and the product was sequenced and compared with the *URA5.3* gene sequence of the wild-type strain. Figure 3a, left shows the results with high-energy neutrons indicating that single-nucleotide substitutions occurred most, accounting for 71.8% of the total analyzed. Next was single-nucleotide insertion, which accounted for 10.3% of the total, followed by single-nucleotide deletion (7.7%), double-nucleotide substitutions (5.1%), double-nucleotide deletions (2.6%), and triple- to single-nucleotide substitutions (2.6%). In the case of 5-FOA resistant strains with thermal neutrons, single-nucleotide substitutions occurred most, accounting for 77.3% of the total analyzed (Fig. 3a, right). Next was single-nucleotide deletion, which accounted for 9.1% of the total, followed by single-nucleotide insertion (4.5%), double-nucleotide deletion (4.5%), and triple- to double-nucleotide substitutions (4.5%). It is important to note that irrespective of the type of neutron beams used, we did not detect any deletion mutant strains lacking the genomic region containing the *URA5.3* gene. These results indicate that the kind of mutation and their occurrence frequencies do not differ much between both neutrons and that single-nucleotide changes abundantly occurred in the neutron irradiations.

Figure 3b shows the characterization of single-nucleotide substitution. In the case of both neutron irradiation conditions, transition and transversion mutations were almost the same. However, transversion mutations tended to have slightly different contents: G:C→T:A and A:T→T:A are abundant in the case of thermal neutron irradiation conditions.

### Change of transcript abundance by exposure to high-energy and thermal neutrons

**Figure 4:**
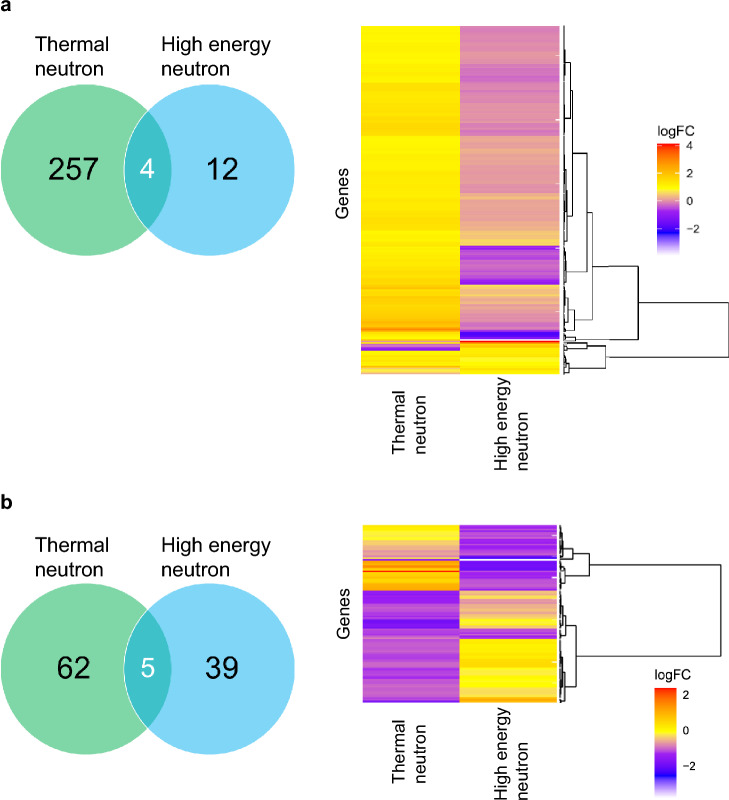
The number and transcriptional patterns of genes up- or down-regulated by thermal or high-energy neutral beam irradiation. Panel (**a**) up-regulated genes by neutral beam irradiation. Panel (**b**) down-regulated genes by neutral beam irradiation. The genes that showed more than two times higher or two times lower transcription in either of the irradiated samples than those in control samples were designated as up- or down-regulated genes (n = 3 for each sample; false discovery rate < 0.05). In the Venn diagrams, the number of the up- or down-regulated genes in each samples is represented. In the heat maps, the genes up- or down- regulated in either of the samples are shown. Log fold change in transcript abundance from control samples is indicated for each gene. logFC: log fold change.

Next, to investigate whether the physiological state of the algal cells changes due to neutron beam irradiation, we analyzed shifts in the transcript levels for each type of beam (Tables S1 and S2). The transcript level of 261 and 16 genes became more than two times higher in the thermal and high-energy neutron-irradiated samples than in the controls, respectively (Fig. 4a). On the other hand, the transcript level of 67 and 44 genes became more than two times lower in the thermal and high-energy neutron-irradiated samples than in the controls (Fig. 4b). Those genes were involved in diverse cellular functions, such as transport and the metabolism system of lipid, carbohydrate, inorganic ion, and amino acid (COG category: E, G, I, and P). All the functional categories (COG and KEGG KO) of the genes up- or down-regulated are listed in Tables S3 and S4.

When we compared the composition of up-regulated genes between the samples, 257 and 12 genes were up-regulated specifically in thermal and high-energy neutron-irradiated samples, respectively (Fig. 4a). While the former 257 genes included the genes involved in 21 COG categories, such as coenzyme transport and metabolism (COG category: H), secondary metabolites biosynthesis, transport, and catabolism (COG category: Q), and cell wall/membrane/envelope biogenesis (COG category: M), the latter 12 genes were associated with only translation (COG category: J), signal transduction mechanism (COG category: T), or post-translational modification, protein turnover, and chaperones (COG category O) (Table S3). Notably, 17 COG categories were up-regulated by only thermal neutron beam (Table S3).

Similarly, 62 and 39 genes were specifically down-regulated in thermal and high-energy neutron-irradiated samples, respectively (Fig. 4b). Annotated functions of the genes specifically down-regulated in thermal or high-energy neutron-irradiated samples shared 12 cellular functions. However, cell cycle control, cell division, and chromosome partitioning (COG category: D), replication, recombination, and repair (COG category: L), and secondary metabolites biosynthesis, transport, and catabolism (COG category: Q) were found to be down-regulated only by thermal neutron irradiation, while cell wall/membrane/envelope biogenesis (COG category: M) was down-regulated only by high-energy neutron irradiation (Table S4).

These results suggested that the transcript levels of multiple cellular functions changed due to beam irradiation and that the manner of these changes varied depending on the type of beam.

### Production of highly-lipid accumulating strain of *E. gracilis* using neutron irradiation

To demonstrate mutant strain production using microalgal species other than *C. merolae*, we conducted neutron irradiation and subsequent mutant screen on *E. gracilis*. The cells of wild-type strain eu029 were irradiated by thermal neutrons and high-energy neutrons over a wide range of intensity (thermal neutrons, 3–25 Gy; high-energy neutrons, 1–54 Gy). Then, the mutagenized cells were subjected to screening of highly-lipid accumulating cells in a normal culture condition following the reported procedure^18^. In the mutant screen, we isolated four mutant strains (B11ZNL, B12ZNL, B13ZNL, and B14ZNL) from irradiation groups of thermal neutrons at 3 and 13 Gy and high-energy neutrons at 5 and 8 Gy.

**Figure 5:**
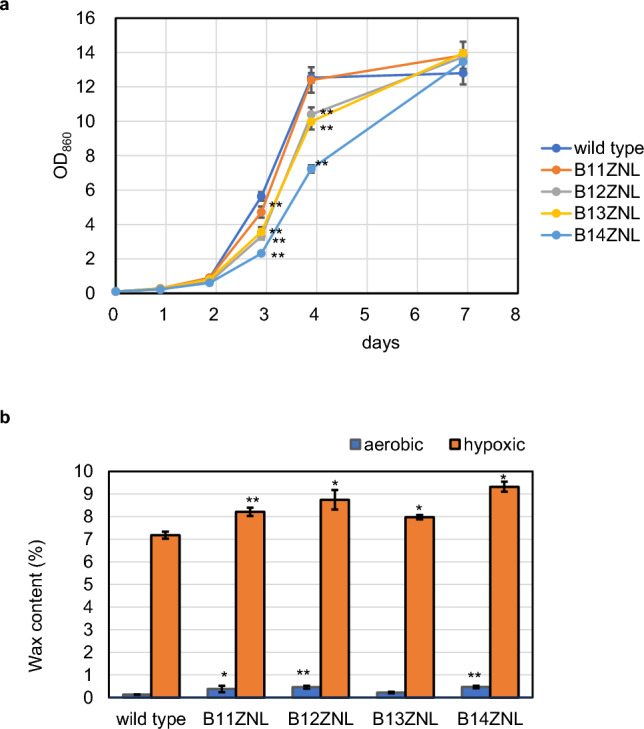
Growth curve and wax-ester accumulation of established *E. gracilis* strains. Panel (**a**) OD_860_ increase in wild-type and four established mutant strains under heterotrophic conditions. n = 3, error bars indicate the standard deviation, p values for post-hoc Dunnett test are shown for the data on days 3 and 4 as follows: **p < 0.01; *p < 0.05. Panel (**b**) wax-ester content in wild-type and four established strains in aerobic and hypoxic conditions. The ratios of the GC-quantified weights of extracted wax esters to those of the dried cell samples subjected to the extraction are shown. n = 3, error bars indicate the standard deviation, p values for post-hoc Dunnett test are shown for both aerobic and hypoxic conditions as follows: **p < 0.01; *p < 0.05.

Based on the measured optical density (OD_860_) of the culture, the established candidate strains proliferated well albeit more slowly than the wild-type (Fig. 5a). Especially, the optical density on days 3 and 4 was significantly lower than that of the wild-type, while no significant difference was detected between the data of the final concentration of the culture on day 7. The wax-ester lipid content after a day of lipid accumulation procedure, hypoxic conditioning, was significantly higher than that of the wild-type with a maximum 1.3-fold increase (Fig. 5b). In addition, the wax-ester yield per culture volume was also significantly higher than that of the wild-type (Fig. S3). Although lipid components other than wax-esters were not quantified in the experiments, since wax-esters comprise most of the lipids under hypoxic conditions, the BODIPY fluorescence increase in the mutants was suggested to be derived from the wax-ester increase. Our results demonstrate that the neutron irradiation is effective for producing mutants of not only *C. merolae* but also other microalgal species including *E. gracilis* whose mutants have been known to be difficult to produce.

## Discussion

In this study, the relationship between absorbed dose and mutation occurrence rate was unequivocally elucidated in algae by using the unicellular red alga *C. merolae*. Furthermore, the types of mutations introduced and the effect of two neutrons, high-energy and thermal neutrons, on the mutation occurrence were analyzed. The obtained analysis results are considered to be fundamental information that can be widely used in algal breeding with neutrons. The irradiation conditions in this study successfully increased the lipid accumulation in *E. galacilis*, four practical strains for jet fuel production by a maximum factor of 1.3.

We hypothesized that high-energy and thermal neutrons affect algal cells differently and tested this hypothesis in this study. The results of the analysis using 5-FOA-resistant strains showed no large differences in the optimum absorbed dose for either irradiation conditions or the type of mutation introduced into the *URA5.3* gene. Note that in the case of high-energy neutrons, the conditions could be examined every 10 Gy. Still, in the case of thermal neutrons, irradiation was carried out at two points every 13 Gy due to the capacity and operation of the irradiation equipment. Although no major differences were observed between the two neutron irradiations concerning mutation introduction, mainly (more than 90%) due to single-base changes, in the case of single-nucleotide substitutions, the composition of transition-type mutations differed between the two neutron irradiation conditions, raising the possibility that the two types of neutron beam irradiation result in different patterns of nucleotide mutation. Clarification of genome-wide detection of mutations will reveal more detailed differences and is an issue to be resolved in the future.

Besides nucleotide base changes by the two types of neutron beam irradiation, transcript levels of 379 genes shifted after irradiation (273 and 106 were up- and down-regulated, respectively; Fig. 4). It is unclear whether these changes in transcript levels are a response to the irradiation itself or are due to some mutation in the transcription regulatory factors or the cis-regulatory elements caused by the irradiation. However, since those up- or down-regulated genes were involved in diverse cellular functions, such as carbohydrate, amino acid, and lipid metabolisms, it should at least be concluded that changes in cellular physiological state can be caused by neutron beam irradiation (Tables S3 and S4). Furthermore, as the genes up-regulated by neutral beam irradiation were likely to be unique to each beam (Fig. 4a) and as 17 COG categories were up-regulated by only the thermal neutron beam (Table S3), patterns of change in cellular metabolism will be affected by the type of beam. Therefore, according to these specific relationships between the up-regulated genes and each neutron beam, we may be able to simply distinguish the type of beam on the basis of patterns of transcript levels. All the possibilities described above should be investigated in future works.

In a previous study, 5-FOA-resistant strains of *C. merolae* were also obtained using spontaneous mutation^13^. As shown in this study, 5-FOA-resistant *C. merolae* strains were also obtained from the cell population exposed to neutron beam irradiations as well as under no-irradiation (0 Gy) conditions (Fig. 2). Compared with the no-irradiation condition, the number of resistant strains increased 14.5-fold and 12.1-fold in the cases of high-energy and thermal neutrons, respectively. Therefore, these results verified that neutron irradiation can induce the *URA5.3* mutation about 12- to 15-fold more frequently than naturally occurring mutations. Neutron irradiation is therefore considered to be a more efficient way of obtaining mutant strains with the desired traits. In *C. merolae*, autofluorescence-based high-throughput isolation of nonbleaching cells using cell-sorters was achieved in a previous study^21^, and its application to the acquisition of mutants with respect to response after nitrogen deprivation is an example. The cell-sorter isolation of high-oil-producing strains of *E. gracilis* using fluorescence derived from BODIPY staining shown in this study is also a good example of neutron-beam-based acquisition of mutants with useful traits.

So far, for breeding algae or plants, mutation induction by exposure to gamma rays has been mainly conducted. For instance, anthocyanin-less strains of Arabidopsis (terrestrial plant) have been obtained by exposing Arabidopsis to gamma rays^22^. That study reported that 25.5% of the total mutations caused by gamma ray exposure was occupied by insertions or deletions of two or more bases (including more than 100 bases), which is higher than the percentage of mutants accounted for by high-energy (9.0%) and thermal (10.3%) neutrons observed in this study. This suggests that mutagenesis by neutron radiation may result in fewer base changes than other radiation types including gamma radiation. The high proportion of changes in a small number of bases caused by neutron irradiations may be advantageous in identifying the causative gene if the mutated gene(s) is highly accumulated. This is another issue to be resolved in the future, as more detailed differences will be revealed by clarifying the genome-wide mode of mutagenesis. Furthermore, another study breeding Chlorella (microalgae) by gamma-ray irradiation reported that a strain that produces a 1.2 to 2.1 times higher amount of lipid than wild-type was obtained through irradiation with 800 Gy of gamma rays^23^. However, under those irradiation conditions, only 10% of the colonies survived. In contrast, under the irradiation conditions in this study, irradiated *C. merolae* grew comparably to the wild-type strain (Fig. 2S). The bred *E. galacilis* strain showed better lipid accumulation than the wild-type, with a maximum increase of 1.3-fold. These data suggest that the efficiency of breeding can be improved using neutron beams rather than gamma rays.

In the study, we focused on breeding techniques that rely on random mutagenesis and subsequent mutant screening and added a new option to enhance mutation introduction to microalgae. In addition to this breeding technique, genome editing^24^ and genetic engineering^25^ are also applied to produce industrially valuable strains. Although the breeding technique based on random mutagenesis is classic, it still offers advantages over others, especially for microalgae, as it can target unknown metabolic pathways, and the strains produced have no legal restrictions on outdoor culture^26^ (Table 1). These characteristics are similar to those of techniques that utilize media additives without relying on mutants to achieve desired effects^27^, while breeding techniques have advantages of not incurring running costs. Each technique has its own advantages and disadvantages, but additive effects may possibly be achieved by combining them. Therefore, continuous improvements in various techniques are desired.

**Table 1:** Comparison of breeding techniques and media additives.

Technique	Method	Advantage	Disadvantage
Random mutagenesis	Chemical mutagen	Inclusion of unknown metabolic pathways as modification targets No restriction on outdoor culture Stable maintenance of strains	Need for establishing effective mutation introduction methods Need for establishing mutant screening methods Accompanying irrelevant mutations
	UV		
	Gamma ray/X-ray		
	Heavy ion beam		
	Neutron (this study)		
Genome editing	ZFN	Planned phenotype improvement Stable maintenance of strains	Need for a deep understanding of metabolic pathways Need for genome sequencing Need for establishing genome editing methods (Legal restrictions on outdoor culture)
	TALEN		
	CRISPR-Cas9		
Genetic engineering	Gene overexpression	Planned phenotype improvement	Need for a deep understanding of metabolic pathways Need for establishing genetic modification methods Instability of strains Legal restrictions on outdoor culture
Media additive	Activator chemicals	Inclusion of unknown metabolic pathways as drug targets No hassle of maintaining strains	Need for establishing chemical screening methods Need for access to chemical libraries Need for running costs in the production
	Inhibitor chemicals		

The advantages and disadvantages of breeding methods, random mutagenesis, genome editing, and genetic engineering, and usage of media additives are summarized.

Our demonstration of mutant production of both *C. merolae* and *E. gracilis* using neutron irradiation suggests that the neutron beam can be used as a general microalgal mutagen. *C. merolae* and *E. gracilis* belong to the eukaryotic supergroups Plantae and Excavata, respectively, indicating their phylogenetic divergence, thus the neutron beam should apply to a wide range of microalgae. The primary difference between neutron beam and heavy-ion beam, which have also been demonstrated to be effective in *E. gracilis* mutant production^17–19^, is the permeability in the water. The attenuation of neutron beams requires a larger amount of water than that of heavy-ion beams, indicating that a larger number of cells can be mutagenized at one time by using a neutron beam. In addition, as shown in our results, a neutron beam causes relatively mild mutations such as a few bases of in-del, indicating that the produced mutants receive a benefit of avoiding severe side effects unlike when using a heavy-ion beam, which typically causes large genomic modification^28^. The unique characteristics of neutron beams clearly suggest the feasibility of applying mutagenic treatments to large populations, potentially enhancing the breeding of algae.

## Material and methods

### Strains and growth conditions

*Cyanidioschyzon merolae* 10D wild-type strain^29^ was grown at 40 °C under continuous white light (50 μmol/m^2^ s) in liquid MA2 medium^30^ at pH 2.5 bubbled with air supplemented with 2% (v/v) CO_2_. The 5-FOA (5-fluoroorotic acid) resistant strains, 5-FOA and uracil, were added to MA2 solid or liquid medium at final concentrations of 0.9 and 0.6 mg/mL, respectively. *Euglena gracilis* wild-type eu029 strain, which is the same as NIES-48, was cultured under constant light (100 μmol/m^2^ s) at 26–29 °C with air supplemented with 5% (v/v) CO_2_ (flow rate of 50 mL/min) in CM3.5 medium^31^.

### Preparation of algal cells exposed to neutron beams

*C. merolae* wild-type cells of stationary phase (OD_750_ > 2–4) were added to 250 or 500 mL volume culture bottles with a final OD_750_ of 0.05 and cultured until the OD_750_ was approximately 0.2. The culture medium was dispensed into 50 mL tubes in 45 mL portions in triplicates per irradiation condition, and each tube was transported to the irradiation facility for neutron irradiation. *E. gracilis* wild-type cells were cultured in 100 mL test tubes containing 50 mL of CM3.5 medium. The cells of log growth phase were diluted to OD_860_ of 0.4 with CM3.5 medium, dispensed into 50 mL tubes in 45 mL portions in triplicates per irradiation condition, and transported to the irradiation facility.

### Irradiation of neutron beams to algal cells

In the case of exposure of high-energy neutrons (carried out at SHI-ATEX Co., Ltd.), the triplicated algal sample tubes were placed 63.5 cm away from the neutron generation target at a current value of 120 µA and irradiated for approximately 15 min per 10 Gy to prepare algal cells irradiated with absorbed doses ranging from 10 to 80 Gy. For each 15-min irradiation, the 50 mL tube was gently shaken up and down 2–3 times to maintain the uniformity of the culture medium. Non-irradiated cells are cells that were not irradiated under the same conditions as the irradiated cells. In the case of thermal neutrons (carried out at Aomori Prefecture Quantum Science Center), the algal cells were placed 62.1 cm away from the neutron-generating target at a current value of 100 μA and irradiated for 5 h per 13 Gy. The jig with the tubes containing algal culture was rotated at 2 rpm during the irradiation. During each irradiation, algae cultured under the same conditions except for neutron irradiation were used as controls (0 Gy irradiation). The irradiated or non-irradiated cells were transferred to vent-capped tissue culture flasks (600 mL volume) and cultured in an air-conditioned room set at 25 °C (approximately 5 μmol/m^2^ s) with shaking (60 rpm) until the dose could be taken out of the radiation-controlled area.

### Isolation of 5-FOA resistant *C. merolae* strains

The number of *C. merolae* cells per 1 mL of the medium after leaving the radiation-controlled area was counted with a hemocytometer. Then, 1 × 10^8^ cells were plated on MA2 solid medium containing 5-FOA and uracil using the top starch method^32^. These plates were incubated at 40 °C under continuous white light (50 μmol/m^2^ s) supplemented with about 5% CO_2_ in AnaeroPack with AnaeroPouch (Mitsubishi Gas Chemical). After 3–4 weeks of incubation, the number of colonies was counted.

### Sequence analysis of 5-FOA-resistant strains

1.5 mL of MA2 medium containing 5-FOA and uracil was added to each well of a 24-well culture plate, and the 5-FOA-resistant colonies (24 and 20 obtained by high-energy neutron and thermal neutron, respectively) were added to each well. The 24-well plates were incubated by enclosing an AnaeroPack with AnaeroPouch under 40 degrees and white light (50 µmol/m^2^ s). The genomic DNA of each strain was isolated from each grown cell by the method described previously^5^ and used as a template for PCR. The DNA fragment containing *URA5.3* (− 540 to + 1685, + 1 as the initiation codon) was simplified by PCR using a set of primers [URA5.3_up_F4 (5′ GGATGAGTCGTACTGAAAGAAGCACAG – 3′) and URA5.3_up_R3 (5′ CCATAGGATCCAGCTCTAGGGACAG – 3′)]. The PCR reaction was carried out in a thermal cycler using DNA polymerase (KOD-ONE, TOYOBO) using the following program: 1 cycle of 98 °C for 2 min, 30 cycles of 98 °C for 10 s and 68 °C for 30 s, and 1 cycle of 98 °C for 5 min. After confirming that the DNA fragments were amplified as a single band by agarose electrophoresis, the PCR product was purified using the Wizard SV Gel and PCR Clean-Up System (Promega). The sequence of each DNA fragment was analyzed by Sanger sequencing using the purified PCR product as a template and the following primers [URA5.3_Sq_F1 (5′ – GGTCATCCGAAGAGAAACCTC – 3′), URA5.3_Sq_F2 (5′ – TTCTGGGAATGGAGCAGCTT – 3′), URA5.3_Seq_F3 (5′ – GACGCTGCGGAATTTGAACT – 3′), and URA5.3_Seq_F4 (5′ – GATTGGCCTCCTGTTAGTCG –3′)]. The resultant results were compared with the *URA5.3* gene sequence registered in *C. merolae* gene database (http://czon.jp/).

### RNA-Seq analysis

*C. merolae* cells after neutron beam irradiation or its control condition were harvested by centrifugation (2800 × g, 5 min, room temperature) for each triplicated tube and the resultant cells were stored at − 80 °C until use. Total RNA was extracted from *C. merolae* cells using RNeasy Plant Mini Kit (QIAGEN) by following the manufacturer’s protocol. One microgram of total RNA was used for library synthesis as follows. Both nuclear and organelle ribosomal RNA was removed using the RiboZero rRNA Removal Kit (Plant Leaf) (Illumina) following the manufacturer’s protocol. Sequencing libraries were prepared using the NEBNext Ultra mRNA library prep kit for Illumina (NEB) with the following modifications. After fragmentation to an average length of 300 bp by high temperature treatment, the first strand cDNA was synthesized using random hexamer primers, followed by the second strand cDNA synthesis. 150 cycles of paired-end sequencing were carried out using the NovaSeq 6000 system by following the manufacturer’s specifications (Illumina, San Diego, USA).

The RNA-Seq reads were trimmed using cutadapt ver. 4.1. with the following parameters: Phred quality score > 20; removal of the read-through adaptor with a match of 10 bases or more; and removal of truncated reads less than 51 nucleotides in length. Trimmed reads were mapped to all the genes in the reference genome of *Cyanidioschyzon merolae* 10D (accession number: GCF_000091205.1 for nucleus, NC_000887.3 for mitochondrion, and NC_004799.1 for chloroplast) using STAR ver. 2.7.9a. The expression level of each gene and log fold changes of transcript abundance against control were quantified by RSEM ver. 1.3.3 using data from triplicated samples for each condition. The genes that showed more than two times higher or two times lower transcription in either of the irradiated samples than those in control samples were regarded as up- or down-regulated genes (false discovery rate < 0.05). The up- and down-regulated genes were classified into COG^33^ category and KEGG KO^34^ by using eggnog-mapper^35^. For a further detailed description, we also referred to *Cyanidioschyzon merolae* Genome Project v3 (http://czon.jp/).

### Isolation of highly-lipid accumulating cells of *E. gracilis*

The mutant screen of *E. gracilis* cells that accumulate higher amounts of lipid than wild-type cells was conducted as reported^18^. Briefly, the cells cultured in KH3.5 medium^36^ were stained by 10 μM of BODIPY^505/515^ in the dark for 5 min and were washed with water by centrifuging the sample at 2000 g for 30 s. Then, the cells were subjected to fluorescence activated cell sorting (FACS) using Moflo XDP (Beckman Coulter) to collect the cells with high fluorescence intensity at 529 nm from the population. The collected cells were cultured in KH3.5 medium and, after proliferation, the cells were subjected to another round of mutant condensation. The collected cells were cultured in KH3.5 medium again and, after proliferation, the cells were next single isolated by FACS. The progenies of isolated cells were characterized for their BODIPY staining phenotype by flow-cytometry with BODIPY^505/515^ staining. The clones that reproducibly showed the phenotype were established as candidates for lipid accumulating strains.

### Evaluation of the highly-lipid containing strains of *E. gracilis*

The characterization of highly-lipid containing strains of *E. gracilis* was conducted by the growth test and lipid quantification as reported^18^. Briefly, the growth test was conducted by culturing in 50 mL of KH3.5 medium in the dark using 100 mL volume conical flasks for 7 days. The growth was evaluated by measuring the cell density as OD_860_. On the 7th day of culture, 10 and 1 mL of the culture were dispensed in two 15 mL tubes and two 1.5 mL tubes, respectively.

They were designated as aerobic and hypoxic samples, respectively. One tube from each size was immediately subjected to centrifugation (2000 g, 2 min), and the other was subjected to hypoxic treatment for 24 h before being centrifuged. The centrifuged samples were immediately frozen, and once all the samples were collected, freeze-dried. The samples collected in the 15 mL tubes were used for weight measurement, while those collected in the 1 mL tubes were subjected to lipid extraction and wax-ester component analysis as described below.

Wax-ester quantification was conducted by extracting lipids with 1 mL of n-hexane, using 3 mm glass beads in a beads crusher MS-100 (Tomy, Japan) at 3000 rpm for 3 min. Subsequently, the solvent was centrifuged at 15 krpm for 5 min to remove debris. Next, 500 μl of the supernatant was transferred to a GC sample vial and subjected to GC analysis using a GC2014 (Shimazu, Japan) equipped with a glass column, Dexsil 300GC 3% Chromosorb W 80/100 mesh AW-DMSC, 1.1 m × 3.2 mm. The analysis conditions involved a temperature increase from 200 to 260 °C at a rate of 5 °C/min, with the temperature kept steady for 25 min. The chromatogram peaks corresponding to C24, C25, C26, C27, C28, C29, C30, C31, and C32, which represent the main components of wax esters, were identified. Subsequently, their concentrations and total weights were quantified by comparing the peak areas with that of C28 wax-ester standard. Furthermore, the ratio of the weight of wax-ester to the dry weight of the cells and the lipid yield per culture medium were calculated.

For statistical analysis, a one-way analysis of variance (ANOVA) and post-hoc Dunnett test were conducted to compare the wild-type with each strain for the growth test on days 3, 4, and 7, as well as for lipid content under both aerobic and hypoxic conditions.

### Supplementary Information

Supplementary Figures.

Supplementary Legends.

Supplementary Tables.

## Data Availability

All data generated or analyzed during this study are included in this published article and its supplementary information files. Information on *C. merolae* genes, such as sequence and description, is based on the *Cyanidioschyzon merolae* database (http://czon.jp/).

## References

[1] Chisti Y (2007). Biodiesel from microalgae. Biotechnol. Adv..

[2] Dismukes GC, Carrieri D, Bennette N, Ananyev GM, Posewitz MC (2008). Aquatic phototrophs: Efficient alternatives to land-based crops for biofuels. Curr. Opin. Biotechnol..

[3] Andrade LM, Andrade CJ, Dias M, Nascimento CAO, Mendes MA (2018). Chlorella and spirulina microalgae as sources of functional foods, nutraceuticals, and food supplements; an overview. MOJ Food Process Technol..

[4] Rizwan M, Mujtaba G, Memon SA, Lee K, Rashid N (2018). Exploring the potential of microalgae for new biotechnology applications and beyond: A review. Renew. Sustain. Energy Rev..

[5] Imamura S, Nomura Y, Takemura T, Pancha I, Taki K, Toguchi K, Tozawa Y, Tanaka K (2018). The checkpoint kinase TOR (target of rapamycin) regulates expression of a nuclear-encoded chloroplast RelA-SpoT homolog (RSH) and modulates chloroplast ribosomal RNA synthesis in a unicellular red alga. Plant J..

[6] Lee TM, Lin JY, Tsai TH, Yang RY, Ng IS (2023). Clustered regularly interspaced short palindromic repeats (CRISPR) technology and genetic engineering strategies for microalgae towards carbon neutrality: A critical review. Bioresour. Technol..

[7] Fukuda S, Hirasawa E, Takemura T, Takahashi S, Chokshi K, Pancha I, Tanaka K, Imamura S (2018). Accelerated triacylglycerol production without growth inhibition by overexpression of a glycerol-3-phosphate acyltransferase in the unicellular red alga *Cyanidioschyzon merolae*. Sci. Rep..

[8] Satoh K, Oono Y (2019). Studies on application of ion beam breeding to industrial microorganisms at TIARA. Quant. Beam Sci..

[9] Bleisch R, Freitag L, Ihadjadene Y, Sprenger U, Steingröwer J, Walther T, Krujatz F (2022). Strain development in microalgal biotechnology—random mutagenesis techniques. Life.

[10] Liu S, Xu J, Chen W, Fu H, Ma LY, Xu H, Xinnian L, Wu M, Ma F (2016). Enhancement of lipid productivity in green microalgae *Chlorella* sp. via fast neutron irradiation. Biomass Bioenergy.

[11] Lone MA, Bigham CB, Fraser JS, Schneider HR, Alexander TK, Ferguson AJ, McDonald AB (1977). Thick target neutron yields and spectral distributions from the 7Li(pd, n) and 9Be(pd, n) reactions. Nucl. Instrum. Methods.

[12] Jeon B, Kim J, Lee E, Moon M, Cho S, Cho G (2022). Target-Moderator-Reflector system for 10–30 MeV proton accelerator-driven compact thermal neutron source: Conceptual design and neutronic characterization. Nucl. Eng. Technol..

[13] Minoda A, Sakagami R, Yagisawa F, Kuroiwa T, Tanaka K (2004). Improvement of culture conditions and evidence for nuclear transformation by homologous recombination in a red alga, *Cyanidioschyzon merolae* 10D. Plant Cell Physiol..

[14] Matsuzaki M (2004). Genome sequence of the ultrasmall unicellular red alga *Cyanidioschyzon merolae* 10D. Nature.

[15] Dragone G, Fernandes BD, Vicente AA, Teixeira JA (2010). Third generation biofuels from microalgae. Curr. Res. Technol. Educ. Top. Appl. Microbiol. Microb. Biotechnol..

[16] Maity JP, Bundschuh J, Chen CY, Bhattacharya P (2014). Microalgae for third generation biofuel production, mitigation of greenhouse gas emissions and wastewater treatment: Present and future perspectives—a mini review. Energy.

[17] Yamada K, Kazama Y, Mitra S, Marukawa Y, Arashida R, Abe T, Ishikawa T, Suzuki K (2016). Production of a thermal stress resistant mutant *Euglena gracilis* strain using Fe-ion beam irradiation. Biosci. Biotechnol. Biochem..

[18] Yamada K, Suzuki H, Takeuchi T, Kazama Y, Mitra S, Abe T, Goda K, Suzuki K, Iwata O (2016). Efficient selective breeding of live oil-rich Euglena gracilis with fluorescence-activated cell sorting. Sci. Rep..

[19] Muramatsu S, Atsuji K, Yamada K, Ozasa K, Suzuki H, Takeuchi T, Hashimoto-Marukawa Y, Kazama Y, Abe T, Suzuki K, Iwata O (2020). Isolation and characterization of a motility-defective mutant of *Euglena gracilis*. PeerJ..

[20] Taki K, Sone T, Kobayashi Y, Watanabe S, Imamura S, Tanaka K (2015). Construction of a URA5.3 deletion strain of the unicellular red alga Cyanidioschyzon merolae: A backgroundless host strain for transformation experiments. J. Gen. Appl. Microbiol..

[21] Takeue N, Kuroyama A, Hayashi Y, Tanaka K, Imamura S (2022). Autofluorescence-based high-throughput isolation of nonbleaching *Cyanidioschyzon merolae* strains under nitrogen-depletion. Front. Plant Sci..

[22] Kitamura S, Satoh K, Oono Y (2022). Detection and characterization of genome-wide mutations in M1 vegetative cells of gamma-irradiated Arabidopsis. PLoS Genet..

[23] Senthamilselvi D, Kalaiselvi T (2023). Gamma ray mutants of oleaginous microalga *Chlorella* sp. KM504965 with enhanced biomass and lipid for biofuel production. Biomass Conv. Bioref..

[24] Jeong BR, Jang J, Jin E (2023). Genome engineering via gene editing technologies in microalgae. Bioresour. Technol..

[25] Muñoz CF, Südfeld C, Naduthodi MIS, Weusthuis RA, Barbosa MJ, Wijffels RH, D'Adamo S (2021). Genetic engineering of microalgae for enhanced lipid production. Biotechnol. Adv..

[26] Trovão M, Schüler LM, Machado A, Bombo G, Navalho S, Barros A, Pereira H, Silva J, Freitas F, Varela J (2022). Random mutagenesis as a promising tool for microalgal strain improvement towards industrial production. Mar. Drugs.

[27] Sun XM, Ren LJ, Zhao QY, Zhang LH, Huang H (2019). Application of chemicals for enhancing lipid production in microalgae-a short review. Bioresour. Technol..

[28] Hirano T, Kazama Y, Ishii K, Ohbu S, Shirakawa Y, Abe T (2015). Comprehensive identification of mutations induced by heavy-ion beam irradiation in *Arabidopsis thaliana*. Plant J..

[29] Kuroiwa T (1998). The primitive red algae *Cyanidium caldarium* and *Cyanidioschyzon merolae* as model system for investigating the dividing apparatus of mitochondria and plastids. Bioessays.

[30] Imamura S, Terashita M, Ohnuma M, Maruyama S, Minoda A, Weber AP, Inouye T, Sekine Y, Fujita Y, Omata T, Tanaka K (2010). Nitrate assimilatory genes and their transcriptional regulation in a unicellular red alga *Cyanidioschyzon merolae*: Genetic evidence for nitrite reduction by a sulfite reductase-like enzyme. Plant Cell Physiol..

[31] Cramer M, Myers J (1952). Growth and photosynthetic characteristics of *Euglena gracilis*. Archiv. Microbiol..

[32] Takemura T, Kobayashi Y, Imamura S, Tanaka K (2019). Top starch plating method for the efficient cultivation of unicellular red alga *Cyanidioschyzon merolae*. Bio Protoc..

[33] Galperin MY, Makarova KS, Wolf YI, Koonin EV (2015). Expanded microbial genome coverage and improved protein family annotation in the COG database. Nucleic Acids Res..

[34] Kanehisa M, Sato Y, Kawashima M, Furumichi M, Tanabe M (2016). KEGG as a reference resource for gene and protein annotation. Nucleic Acids Res..

[35] Cantalapiedra CP, Hernández-Plaza A, Letunic I, Bork P, Huerta-Cepas J (2021). eggNOG-mapper v2: Functional annotation, orthology assignments, and domain prediction at the metagenomic scale. Mol. Biol. Evol..

[36] Koren LE, Hutner SH (1967). High-yield media for photosynthesizing *Euglena gracilis* Z. J. Protozool..

