# Supplementary figures and images for: Optimal conditions of algal breeding using neutral beam and applying it to breed Euglena gracilis strains with improved lipid accumulation
Source: Sci Rep. 2024 Jul 3;14:14716. doi: 10.1038/s41598-024-65175-1 (PMC11222385; doi:10.1038/s41598-024-65175-1)

Figure S1

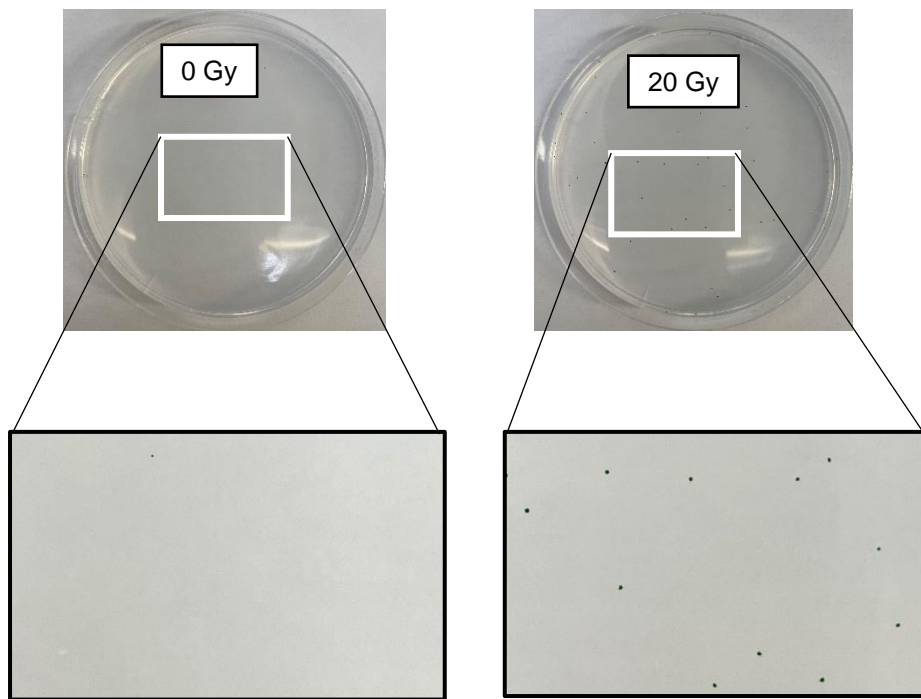

**a**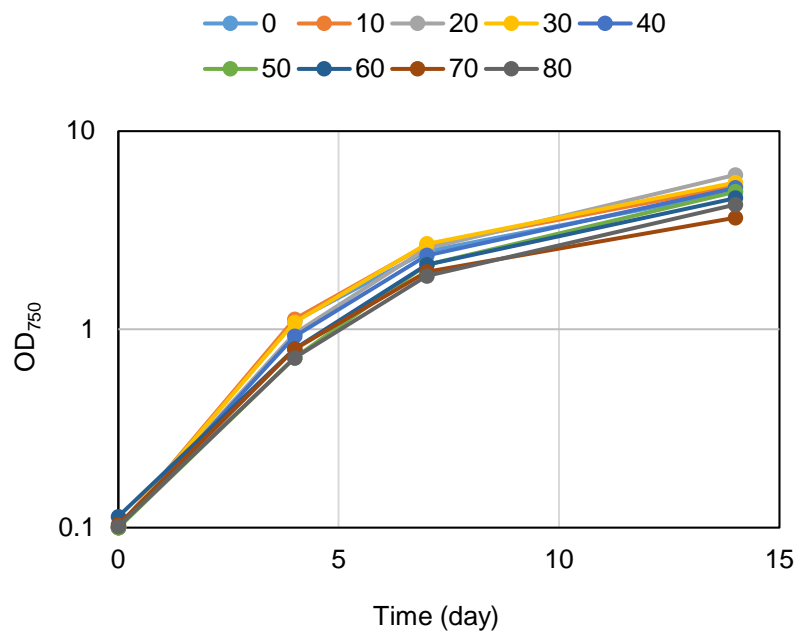**b**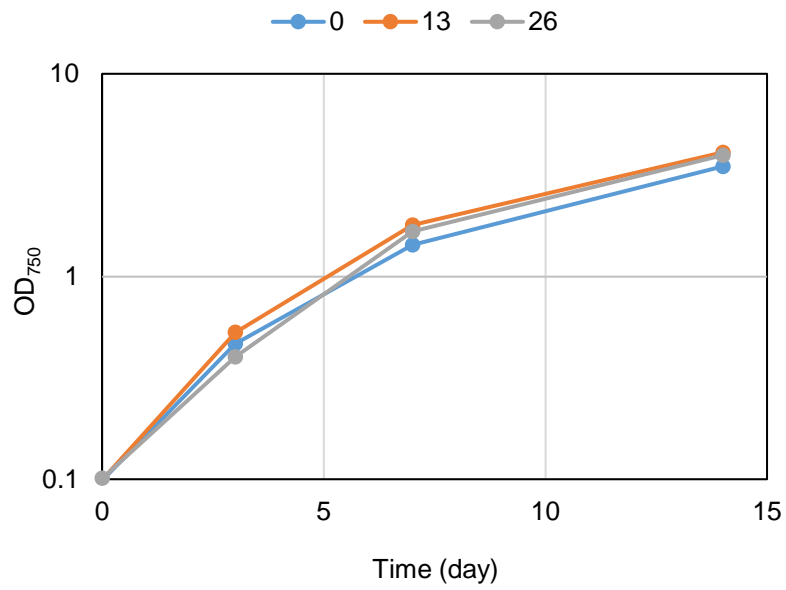

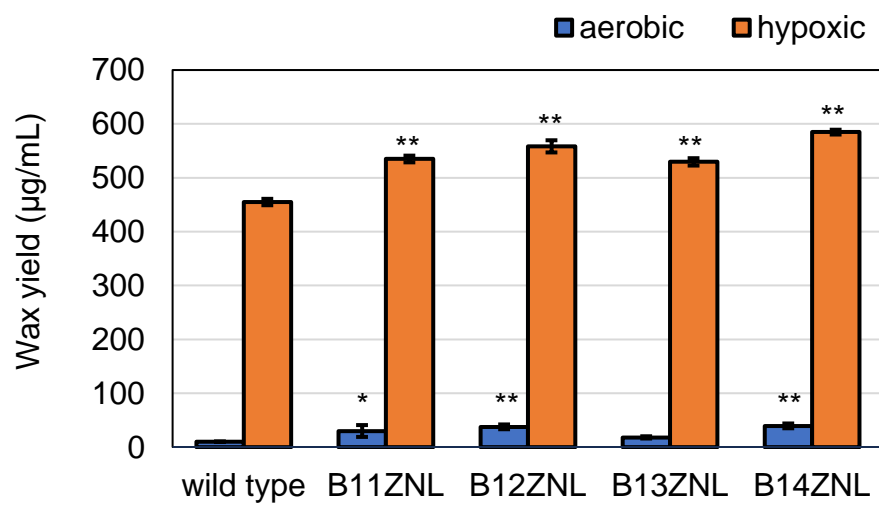

Supplement: Supplementary file 1 — Supplementary Figures. [file 41598_2024_65175_MOESM1_ESM.pdf]
