# Supplementary material for: Optimal conditions of algal breeding using neutral beam and applying it to breed Euglena gracilis strains with improved lipid accumulation
Source: Sci Rep. 2024 Jul 3;14:14716. doi: 10.1038/s41598-024-65175-1 (PMC11222385; doi:10.1038/s41598-024-65175-1)
Supplement: Supplementary file 2 — Supplementary Legends. [file 41598_2024_65175_MOESM2_ESM.docx]

**Supplementary Information**

**Figure S1. 5-FOA-resistant colonies grown on solid medium.**

Photographs of plates after several weeks of plating high-energy neutron-irradiated cells on solid medium containing 5-FOA. Plate images with unirradiated (0 Gy, left) and 20 Gy (right) irradiated cells are shown as representative examples (Top).

Images of a portion of the plate magnified are shown respectively (bottom).

**Figure S2. Growth of *C. merolae* irradiated to neutron beams.**

Panel a. Growth of *C. merolae* cells irradiated to high-energy neutron beams in MA2 media. Growth of each irradiation condition (indicated on the top, the unit is Gy) was monitored by measuring the optical density at 750 nm (OD_750_). Panel b. Growth of *C. merolae* cells irradiated to thermal neutron beams in MA2 media. The others are the same as in panel a.

**Figure S3. Wax-ester yield of established *E. gracilis* strains.**

Wax-ester yield in wild-type and four established strains’ culture in aerobic and hypoxic conditions. The values are the GC-quantified weights of extracted wax esters divided by the volume of the culture containing the cells subjected to the extraction. n=3, error bars indicate the standard deviation, p values for post-hoc Dunnett test are shown for both aerobic and hypoxic conditions as follows: **p < 0.01; *p < 0.05.
